# Trehalose: Neuroprotective Effects and Mechanisms—An Updated Review

**DOI:** 10.3390/neurosci5040032

**Published:** 2024-10-12

**Authors:** Borislav Sevriev, Simeonka Dimitrova, Gabriela Kehayova, Stela Dragomanova

**Affiliations:** 1 Faculty of Pharmacy, Medical University of Varna “Prof. Dr. Paraskev Stoyanov”, 9000 Varna, Bulgaria; borislav.sevriev@gmail.com; 2 Department of Pharmacology, Toxicology and Pharmacotherapy, Faculty of Pharmacy, Medical University of Varna “Prof. Dr. Paraskev Stoyanov”, 9000 Varna, Bulgaria; simeonka.dimitrova@mu-varna.bg (S.D.); gabriela.kehayova@mu-varna.bg (G.K.)

**Keywords:** trehalose, neuroprotection, apoptosis, neuroinflammation, oxidative stress

## Abstract

Trehalose is a naturally occurring disaccharide that has recently gained significant attention for its neuroprotective properties in various models of neurodegeneration. This review provides an overview of available experimental data on the beneficial properties of trehalose for central nervous system pathological conditions. Trehalose’s impact on neuronal cell survival and function was also examined. As a result, we identified that trehalose’s neuroprotection includes autophagy modulation as well as its capability to stabilize proteins and inhibit the formation of misfolded ones. Moreover, trehalose mitigates oxidative stress-induced neuronal damage by stabilizing cellular membranes and modulating mitochondrial function. Furthermore, trehalose attenuates excitotoxicity-induced neuroinflammation by suppressing pro-inflammatory cytokine release and inhibiting inflammasome activation. A possible connection of trehalose with the gut–brain axis was also examined. These findings highlight the potential therapeutic effects of trehalose in neurodegenerative diseases. According to the conclusions drawn from this study, trehalose is a promising neuroprotective agent as a result of its distinct mechanism of action, which makes this compound a candidate for further research and the development of therapeutic strategies to combat neuronal damage and promote neuroprotection in various neurological diseases.

## 1. Introduction

Neurodegenerative diseases (NDs) present a growing and significant health challenge due to their severe and chronic impact on affected individuals. A defining pathological feature of many late-onset NDs, often referred to as proteinopathies, is the aberrant accumulation of misfolded proteins. These conditions encompass a range of disorders including Parkinson’s disease (PD), Alzheimer’s disease (AD), tauopathies, amyotrophic lateral sclerosis (ALS), and polyglutamine (polyQ) expansion disorders such as Huntington’s disease (HD) and various spinocerebellar ataxias (SCAs) such as SCA3 [1].

Misfolded proteins in these neurodegenerative conditions can result from various biological processes. For instance, in AD, the tau protein becomes hyperphosphorylated, leading to the formation of neurofibrillary tangles. In PD, α-synuclein misfolding results in Lewy body formation. Genetic mutations contribute to these misfolding events as well. For example, mutations in the huntingtin gene lead to polyglutamine expansions in HD, while α-synuclein mutations are implicated in PD. Additionally, prion diseases involve the misfolding of the PrPC protein, and ALS is associated with the misfolding of superoxide dismutase 1 (SOD1) and TDP-43.

These misfolded proteins tend to aggregate into β-sheet-rich structures, forming plaques or tangles that disrupt cellular functions. The accumulation of such aggregates overwhelms the cell’s protein quality control mechanisms, which include the ubiquitin-proteasome system and chaperone-mediated autophagy. These systems are responsible for degrading and recycling damaged proteins and organelles. When these systems fail or are impaired, the result is an accumulation of toxic aggregates, which further disrupt cellular processes and contribute to neuronal damage and death.

Recent studies have highlighted a critical link between impaired autophagy and the progression of neurodegenerative diseases [2]. Autophagy is a cellular process essential for maintaining homeostasis by clearing damaged proteins and organelles. Enhancing autophagy has been proposed as a therapeutic strategy to alleviate neuropathology and neurodegeneration, leading to considerable interest in agents that can modulate this pathway [3].

Another prominent feature of disorders like AD, PD, and ALS is the pervasive presence of oxidative stress, which is implicated in the dysfunction and eventual apoptosis of neuronal cells, thereby contributing significantly to disease pathogenesis [4].

Oxidative stress results from the excessive production of reactive oxygen species (ROS), including hydrogen peroxide (H_2_O_2_), nitric oxide (NO), superoxide anion (O_2_⁻), and the highly reactive hydroxyl radical (·OH). These ROS can inflict substantial damage on cellular components, including lipids, proteins, and nucleic acids. The brain’s vulnerability to oxidative damage is exacerbated by its high oxygen consumption, relatively low levels of endogenous antioxidants, and limited regenerative capacity [4].

The imbalance between ROS production and the brain’s antioxidant defenses leads to a cascade of oxidative modifications, including protein carbonylation, lipid peroxidation, and DNA oxidation. These modifications disrupt cellular function, trigger inflammatory responses, and promote neuronal cell death. Although the exact role of oxidative stress in neurodegenerative diseases remains a subject of ongoing research, accumulating evidence suggests that it is a crucial factor in disease progression. As a result, a variety of therapeutic strategies are being developed to target this phenomenon.

**Figure 1:**
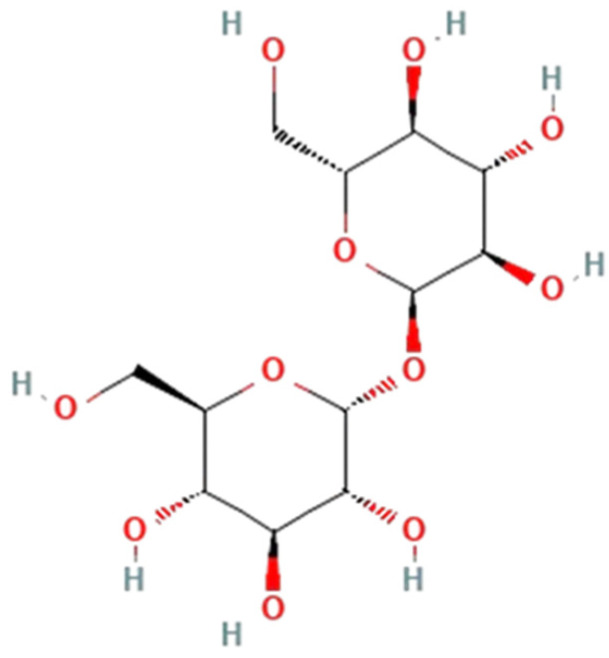
Structure of trehalose [5].

Trehalose (Figure 1), a nonreducing disaccharide composed of two glucose molecules connected by an α,α-1,1-glycosidic bond, has emerged as a promising candidate in the context of neuroprotection.

Found across a diverse range of organisms including bacteria, yeast, fungi, insects, invertebrates, and plants, trehalose serves various biological functions, including energy storage and a source of carbon [6]. Recent research has identified trehalose as an effective autophagy inducer that may offer protection against neurodegenerative pathology. By activating autophagy, trehalose promotes the clearance of misfolded proteins and damaged organelles, which could potentially alleviate the accumulation of toxic aggregates [1,7,8,9].

Furthermore, trehalose’s neuroprotective effects extend beyond its role in autophagy. Evidence suggests that trehalose also reduces oxidative stress, stabilizes cellular proteins, and may influence gut–brain signaling pathways [10,11,12]. These combined actions contribute to its therapeutic potential, making it a compelling candidate for further investigation as a treatment for neurodegenerative diseases.

This review thoroughly evaluates existing studies on trehalose, organizes the findings, and discusses the possible neuroprotective mechanisms through which trehalose might ameliorate destructive cellular events associated with neurodegenerative diseases.

## 2. Materials and Methods

We conducted a thorough search of several academic databases, including Scopus, MEDLINE/PUBMED, ResearchGate, and ScienceDirect. The search utilized a range of keywords, including “trehalose”, “neuroprotection”, “autophagy”, “biological”, “oxidative stress”, “gut–brain”, “inducing”, “Huntington’s disease”, “Parkinson’s disease”, “Alzheimer’s disease”, “amyotrophic lateral sclerosis”, “tauopathies”, “cellular stress responses” and “neurodegenerative diseases”, to ensure a comprehensive search.

Each of the individual topics mentioned above was searched separately to capture a broad spectrum of relevant literature. We included studies published in English from 1 January 1989 to 1 July 2024. To further refine our search, we manually reviewed some of the references of selected articles to identify additional relevant studies.

We excluded studies where trehalose was investigated for effects not directly related to the themes of this article. The comprehensive search process resulted in a significant number of studies, which were then rigorously reviewed and analyzed based on their relevance to the specific topics of interest. The insights and outcomes from this detailed review are presented in the following sections, categorized by their relevance and contributions to the subject matter.

During the preparation of this manuscript, the authors used the AI model “ChatGPT-4” as a paraphrasing tool and language-enhancing tool to ensure clarity and coherence in the presentation of the material.

## 3. Mechanisms of Neuroprotection

**Figure 2:**
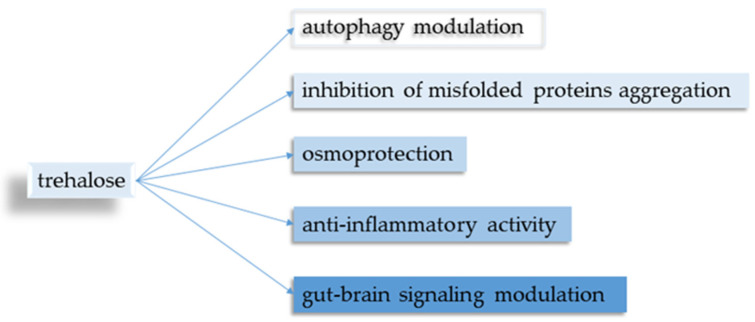
Biological properties of trehalose.

Five main points regarding trehalose and its neuroprotective properties were identified (Figure 2).

### 3.1. Autophagy Modulation

Trehalose has been increasingly recognized for its role in modulating autophagy, a critical process for the degradation and recycling of damaged proteins and organelles. Numerous studies have documented the connection between the substance and its autophagy-modulating properties, demonstrating that trehalose can enhance autophagic activity and improve cellular homeostasis. These findings suggest that this disaccharide holds promise as a therapeutic agent in diseases where autophagy is compromised. The induction of autophagy by trehalose is well-established in the scientific literature [9,13,14,15,16,17,18,19]. The main ways in which it can influence the autophagy include:

#### 3.1.1. mTOR Pathway Inhibition

The mechanistic target of the rapamycin (mTOR) signaling pathway serves as a crucial regulator that integrates environmental stimuli with cellular responses. It exerts direct control over essential cellular processes including translation initiation, transcriptional activity, autophagy, metabolism, and organelle biogenesis. These functions all together contribute to cellular homeostasis and the adaptation of organisms.

In the context of neurobiology, mTOR signaling is intricately involved in diverse facets of brain function. It orchestrates neural progenitor cell proliferation and differentiation, the formation and maintenance of synaptic connections, activity-dependent synaptic plasticity, and the regulation of complex behavioral patterns such as feeding behavior, sleep–wake cycles, and circadian rhythms.

Pathological shifts in mTOR signaling are implicated in a spectrum of monogenic disorders and contribute significantly to the pathogenesis of neurodegenerative conditions and neuropsychiatric disorders [20]. As a result of that, strategies aimed at pharmacologically modulating the mTOR pathway hold substantial promise for therapeutic approaches in neurological and psychiatric disorders, justifying further investigation and development.

AMP-activated protein kinase (AMPK) is an important factor in cellular energy balance and has diverse regulatory effects on several metabolic pathways. One of AMPK’s primary jobs is to activate autophagy. The autophagy itself is a cellular disintegration and recycling process. This occurs through both direct and indirect processes involving Unc-51 Like Autophagy Activating Kinase 1 (ULK1) [21]. AMPK directly phosphorylates ULK1, thereby initiating the autophagy process. Additionally, AMPK has an indirect impact on autophagy. Secondly, AMPK indirectly activates ULK1 through the inhibition of mTORC1, which normally phosphorylates ULK1 and inhibits its interaction with AMPK. This coordinated regulation of ULK1 and mTORC1 facilitates the removal of damaged mitochondria and preserves mitochondrial integrity under conditions of nutrient starvation. AMPK’s functions guarantee that cellular components are effectively recycled and destroyed under energy-stressed situations [21].

AMPK also plays an important role in stimulating mitochondrial biogenesis. It controls the expression of peroxisome proliferator-activated receptor gamma coactivator gamma 1-alpha (PGC-1α), a transcription coactivator that promotes the transcription of genes involved in mitochondrial function and biogenesis. By enhancing PGC-1α activity, AMPK ensures an increase in mitochondrial number and improves mitochondrial function, which is important for maintaining cellular energy balance [21].

In addition, AMPK also activates the cellular antioxidant defense mechanisms. These effects are reportedly achieved by upregulating the expression of antioxidant genes and enhancing the activity of antioxidant enzymes [21]. This function is vital for protecting cells against oxidative stress, which can damage cellular components and impair function.

Trehalose is shown to possess the ability to inhibit the mTOR pathway through various mechanisms, presented below:

- Role of AMPK

Trehalose has been reported to activate AMPK, which as a result can inhibit the mTOR and lead to the activation of autophagy. It does so by creating a cellular environment that can mimic acute stress. DeBosch BJ et al. (2016), using the HEK293 cell line in their study, stated that trehalose may block the entry of glucose and fructose via the GLUT family of transporters into hepatocytes [22]. Thereby, trehalose indirectly leads to the inhibition of glycolysis and the citric acid cycle, eventually leading to ATP deficiency. AMPK is usually activated when cellular ATP levels fall and AMP/ATP or ADP/ATP rise, indicating the low energy state of the cell. This stress is sensed by the cellular machinery and AMPK and ULK1 are activated. The result is the initiation of the autophagy process [22].

However, the precise mechanisms by which cells absorb trehalose are not yet fully understood. SLC2A8 (GLUT8) has been identified as a transporter of trehalose in mammals, facilitating its uptake and triggering autophagy in hepatocytes. However, in neuronal cells such as N2A neuroblastoma cells, the accumulation of LC3-II, an indicator of autophagy, in response to trehalose is not dependent on SLC2A8. This discrepancy is due to the absence of SLC2A8 on the plasma membrane of neuronal cells [11].

These findings suggest that the process of trehalose uptake may differ between neuronal cells and hepatocytes, potentially resulting in varying effects on the autophagic pathway. In addition, in contrast to observations in animal models where trehalose administration induces autophagy in the brain, experiments conducted directly on cultured cells, including neurons, indicate that trehalose may inhibit rather than stimulate autophagy [11].

Furthermore, a recent study by Bohan Li et al. (2021) using an NSC34 cell line demonstrated that trehalose significantly increases the phosphorylation of AMPK and ULK1, as well as SIRT1 levels, further implementing the role of AMPK. The authors draw the conclusion that trehalose treatment was found to significantly enhance the survival rate of motor neurons by promoting autophagy and inhibiting apoptosis [23].

- Interference with mTORC1

It has been suggested that trehalose might interfere with the upstream regulators of mTORC1 in porcine oocytes [24]. This includes Tuberous sclerosis proteins 1 and 2, also known as TSC1 (hamartin) and TSC2 (tuberin), which negatively regulate mTORC1 activity. The increased activity of TSC1/TSC2 leads to the inhibition of mTORC1 activity. Since mTORC1 is a major negative regulator of autophagy, its inhibition promotes autophagy.

By enhancing the activity of these regulatory proteins, trehalose can suppress the activation of mTORC1, ultimately leading to the promotion of autophagy.

Trehalose is also known to induce cellular stress responses in vitro, including endoplasmic reticulum stress in NSC34 cells [16].

The accumulation of trehalose in the lysosomes leads to modest stress and disrupts their ability to maintain proper acidification in mice macrophages [9]. This disruption results in the inactivation of mTORC1, a key regulator that normally suppresses TFEB. With the removal of this suppressive phosphorylation, TFEB is released from its inhibited state. Once liberated, TFEB translocates to the nucleus, where it initiates a transcriptional response that governs the autophagy–lysosome pathway. This response is crucial for maintaining cellular homeostasis by enhancing the expression of genes involved in autophagy and lysosomal function. (See also Section 3.1.3).

#### 3.1.2. mTOR Independent Autophagy Induction

An in vitro study by Sarkar et al., 2007 using human neuroblastoma cells (SKN-SH) and African green monkey kidney cells (COS-7) demonstrated that trehalose induces autophagy independently of the mTOR pathway [13]. The authors point to two possible mechanisms. The first one suggests that the two pathways may be truly independent and can also act on different components of the autophagy machinery. The second hypothesis is that trehalose may act on a middle component in the pathway between mTOR and autophagy. However, they concluded that it is impossible to test the second hypothesis without knowing what these components are, as they are not currently considered to be involved in mammalian autophagy processes.

Trehalose treatment has been shown to induce autophagy in HaCaT cells through an mTOR-independent mechanism as well [19]. In this study, Chen et al. confirmed that the activities of both mTORC1 and mTORC2 were not influenced in trehalose-treated HaCaT cells. They observed that the phosphorylation levels of mTOR and the regulatory-associated protein of mTOR (Raptor), an essential component of the mTORC1 complex, remained unchanged following trehalose treatment. This indicates that the autophagic response induced by trehalose occurs via a pathway independent of mTOR signaling.

Another study conducted by Del Bello et al. (2022) concluded that neither the canonical inhibition of mTOR nor the activation of AMPK was observed in the U373-MG and T98G cell lines, regardless of the level of autophagy attained. These noteworthy findings support the notion that the autophagy induced by trehalose functions independently of both mTOR inhibition and AMPK activation [25].

#### 3.1.3. TFEB Activation

Transcription factor EB or TFEB acts as a key master gene in the control of lysosomal biogenesis [26]. It encodes transcription factors that carefully regulate the expression of various lysosomal hydrolases and membrane proteins as well as genes involved in the autophagy process [26,27]. Under conditions such as nutrient deprivation or abnormal lysosomal accumulation, which often occur in lysosomal storage diseases, TFEB undergoes significant translocation from the cytoplasm to the nucleus. This translocation leads to the activation of target genes, thereby initiating a cascade of gene expression aimed at combating these cellular stressors [26,27]. Furthermore, the experimental overexpression of TFEB in cultured HeLa cells demonstrated its potent ability to enhance lysosomal biogenesis [26]. This overexpression promotes not only lysosome formation but also exocytosis, the process by which cells expel waste products. In addition, it stimulates autophagy, a key cellular process for breaking down and recycling damaged cellular components. Through these mechanisms, TFEB plays a key role in maintaining cellular homeostasis and responding to metabolic stress [26,27,28]. Trehalose can activate TFEB, which then is translocated to the nucleus and promotes the expression of genes involved in autophagy and lysosomal function [9]. This increases the cells’ ability to degrade and recycle cellular components.

Trehalose activates TFEB through several mechanisms related to its role in cellular stress response and autophagy induction.

First, trehalose affects nutrient-sensing pathways that regulate TFEB. Under nutrient-rich conditions, TFEB is phosphorylated by mTORC1 and secreted into the cytoplasm [9]. The disaccharide promotes autophagy and alters nutrient signaling, thereby reducing mTORC1 activity and leading to TFEB dephosphorylation and nuclear translocation in isolated mice macrophages [9]. In the cell nucleus, TFEB activates genes involved in lysosomal biogenesis and autophagy.

Second, trehalose has been shown to affect the calcium signaling pathways, which are important for TFEB activation [16].

A trehalose-activated pathway was identified in mouse motor neurons, initiated by the transient expansion and subsequent permeabilization of lysosomal membranes under osmotic stress [16]. This osmotic stress results in the release of Ca^2+^ ions from the destabilized lysosomes. The released Ca^2+^ ions then activate PPP3CB, a calcium-dependent and calmodulin-stimulated protein phosphatase. Activated PPP3CB dephosphorylates the transcription factor EB (TFEB), which is essential for its translocation to the nucleus, where TFEB can regulate the gene expression involved in lysosomal biogenesis and autophagy.

Third, the activation of transcription factors such as TFEB promotes the expression of autophagy and lysosome-related genes [9]. The activation of TFEB leads to the increased synthesis of lysosomal proteins, including those involved in chaperone-mediated autophagy. Chaperone-mediated autophagy is a selective type of autophagy in which certain proteins are transported directly across the lysosomal membrane for degradation. This is particularly important for removing misfolded or damaged proteins and is beneficial in neurodegenerative diseases.

A study demonstrated that the early administration of trehalose in zebrafish reduced neuronal excitability, lowered the frequency of spontaneous seizures, and improved motor impairments, suggesting its potential therapeutic value for conditions such as Lafora disease in children [29]. This research revealed high levels of TFEB mRNA in laforin-deficient larvae, a finding that aligns with observations in other neurodegenerative diseases. This increased TFEB expression might indicate a compensatory mechanism aimed at overcoming compromised lysosomal function via autophagy. However, TFEB may remain sequestered in the cytosol in its inactive form due to mTOR-mediated phosphorylation, which blocks its nuclear translocation and the activation of autophagy-related genes. The authors hypothesize that trehalose might activate TFEB and facilitate its movement into the nucleus, thereby promoting autophagy.

Trehalose’s autophagy involvement is summarized in Table 1.

### 3.2. Inhibition of Protein Clustering

Protein aggregation is a hallmark of many neurodegenerative conditions [24]. Trehalose has been found to inhibit the aggregation of misfolded proteins. By preventing protein aggregation, trehalose can reduce cellular toxicity, including neurotoxicity.

A layer of water molecules that contribute to stability surrounds proteins in their native state. This hydration shell helps maintain the protein’s three-dimensional structure and facilitates proper folding.

In their study, Carpenter and Crowe (1989), using infrared spectroscopy, showed that trehalose can replace some of these water molecules through hydrogen-bonding interactions with both protein and water in bovine serum albumin [30]. The observed process leads to the stabilization of the protein structure under conditions where the natural hydration shell may be disrupted. These hydrogen bonds inhibit abnormal intermolecular interactions that lead to protein aggregation. It is still uncertain whether and how this interaction contributes to the stabilization of proteins [30].

Heat shock and oxidative stress can lead to protein unfolding, exposing hydrophobic regions that promote aggregation.

A comprehensive study by Singer and Lindquist from 1998 provided significant insights into the protective role of trehalose in cells exposed to heat stress [31]. Their research, which included both in vivo and in vitro experiments, highlighted the crucial function of trehalose in stabilizing proteins at elevated temperatures. By employing two different temperature-sensitive reporter proteins, they observed that enzymes retained a higher degree of activity during heat shock in cells that were able to produce trehalose.

An additional and critical finding from their study was the ability of trehalose to suppress the aggregation of proteins that had already undergone denaturation. This indicates that trehalose not only helps in maintaining protein stability but also prevents further damage to already affected proteins.

The researchers further demonstrated the importance of rapidly degrading trehalose after the end of the heat shock. They found that when the unfolded luciferase, one of the reporter proteins they used, is removed from the trehalose environment, it could effectively be refolded by molecular chaperones. However, if the concentration of trehalose remains high, it interferes with the refolding process, thereby hindering the chaperones from renaturing the protein. Therefore, the study emphasizes the need for active trehalase enzymes in the environment to degrade trehalose after the heat stress is alleviated. This allows the molecular chaperones to efficiently refold denatured proteins and restore their normal functions. The findings of Singer and Lindquist (1998) provide a better understanding of the double role of trehalose in the protection and management of protein folding during and after thermal stress [31].

Trehalose forms a crystalline structure upon drying that traps and stabilizes proteins, preventing denaturation and aggregation [32]. In this crystalline form, disaccharide molecules are arranged in an amorphous, non-crystalline matrix. Trehalose is effective in neutralizing and stabilizing biomolecules, including proteins and membranes, by forming a glass [32]. This immobilization process helps proteins maintain their functional state and not aggregate or denaturate, which often occurs under osmotic stress and dehydrating conditions.

As described above trehalose enhances autophagy, aiding in the clearance of misfolded and aggregated proteins [7,16]. This can reduce the load of misfolded proteins, decreasing the chances of further aggregation.

Tanaka et al. (2004) demonstrated that trehalose inhibits aggregation of proteins by interacting with expanded polyglutamines using a mouse model of HD [33]. They also indicated that trehalose has the potential to inhibit protein aggregation at the initial stage of aggregate formation. This inhibition occurs through the increased stability of polyglutamine-containing proteins, thereby preventing the early onset of aggregation.

Sarkar et al. (2007) showed that trehalose attenuates polyQ-mediated accumulation and cytotoxicity, while also facilitating the clearance of soluble mutant huntingtin [13]. In their study, trehalose significantly reduced aggregation and cell death associated with EGFP-tagged huntingtin exon 1 containing 74 polyQ repeats (EGFP-HDQ74) in both COS-7 cells (non-neuronal) and SK-N-SH cells (neuronal precursor). This effect is specific to trehalose, as the authors did not observe similar results with other disaccharides such as sucrose, the trisaccharide raffinose, or the sugar alcohol sorbitol. These findings suggest that the protective properties of trehalose are unique and not shared by other structurally related sugars. Furthermore, initially, it was thought that trehalose’s ability to stabilize partially unfolded mutant proteins with expanded polyQ regions accounted for its protective effects. However, the authors revealed a different underlying mechanism. They found that trehalose primarily stimulates autophagy, which enhances the clearance of mutant proteins. This conclusion is supported by their observation that the effectiveness of trehalose in reducing aggregates of mutant huntingtin was completely lost when the autophagy process was inhibited.

Through the mechanisms that are mentioned above, trehalose effectively inhibits protein accumulation and promotes protein stabilization both in vivo and in vitro, which is beneficial in the context of neurodegenerative diseases and other conditions associated with protein misfolding and aggregation (Table 2).

### 3.3. Osmoprotective Effect

Neurons are very sensitive to changes in osmotic pressure due to their unique membrane properties and rapid metabolism. Maintaining near constant osmotic pressure is important for maintaining neuronal activity and preventing cell swelling or shrinkage, which can disrupt cellular processes and lead to cell death [35]. Osmotic changes can lead to a number of complications, including brain edema. Nerve edema and inflammation can affect nerve function by increasing intracranial pressure, reducing blood flow, and causing nerve damage. Osmotic stress has been linked to a variety of neurological disorders, including stroke, traumatic brain injury, and neurodegenerative diseases. The disruption of osmotic pressure increases neuronal damage and contributes to disease progression by disrupting the cellular and molecular processes required for neuronal survival.

Trehalose exerts an osmoprotective effect through several mechanisms that help cells withstand osmotic stress:

#### 3.3.1. Membrane Stabilization

In a glassy state, trehalose molecules arrange themselves in an amorphous, non-crystalline matrix. When trehalose forms a glass, it effectively immobilizes and stabilizes biomolecules, including proteins and membranes. This immobilization prevents the biomolecules from undergoing denaturation or aggregation that typically occurs under dehydrating conditions. The vitrification process helps proteins maintain their functional conformations under osmotic stress.

Trehalose interacts with phospholipid bilayers of mammalian cells by forming hydrogen bonds between the hydroxyl groups and the phosphate groups of membrane phospholipids, as described by Crowe et al. (1992) [36]. This molecular interaction strongly stabilizes the bilayer structure, especially under conditions of osmotic stress such as dehydration or exposure to hyperosmotic solutions in mammalian cells [37] and in a strain of wild-type Saccharomyces cerevisiae [38]. By integrating within the lipid bilayer, trehalose reduces membrane permeability, which is important to avoid phase transitions, and the formation of non-bilayer structures, which are usually less stable, can compromise membrane integrity and function. This stabilizing mechanism allows cell membranes to maintain their structure and function even under adverse conditions. The protective function of trehalose is important for cell strength, allowing cells to resist and adapt to various stresses, thereby protecting cell health and vitality in vitro in a dry state [39].

#### 3.3.2. Oxidative Stress Reduction

Trehalose has antioxidant properties that help reduce the oxidative damage associated with osmotic stress. Benaroudj et al. (2001) used bacterial strains tracking their resistance to H_2_O_2_ in the presence of H_2_O_2_/FeCl_3_ [40]. The results showed a significant resistance to reactive oxygen species. The suggested mechanism by which trehalose enhances resistance to oxidative stress was proposed to be the ability of the sugar to scavenge free radicals.

Through these mechanisms, trehalose acts as an effective osmoprotectant, helping cells survive and function under conditions of osmotic stress, such as dehydration, high salinity, or rapid changes in environmental water availability.

### 3.4. Anti-Inflammatory Properties

Trehalose reduces inflammation in human corneal epithelial cells in a hyperosmotic stress state through various mechanisms that modulate immune responses and cellular signaling pathways [41]. The key mechanisms by which trehalose exerts its anti-inflammatory effects are as follows:

#### 3.4.1. Modulation of Inflammatory Mediators: Trehalose’s Role in Suppressing Pro-Inflammatory Cytokines

Studies have concluded that trehalose has the ability to significantly inhibit the production of pro-inflammatory cytokines, thereby attenuating the inflammatory response in isolated and cultured macrophages [42,43].

Interleukin-1β (IL-1β) is crucial for orchestrating inflammatory responses through its binding to IL-1 receptors (IL-1R), which are expressed in various cell types [44]. It is synthesized in response to the activation of inflammasomes, which are protein complexes that activate caspases. This activation process is pivotal in enhancing immune reactions and promoting short-term inflammation as part of the body’s defense against pathogens. IL-1β also plays a significant role in shaping adaptive immunity, contributing to the overall immune response to infections and other challenges to the body’s homeostasis.

Taya et al. (2009) found that stimulated mouse peritoneal macrophages, capable of producing IL-1β, showed a significant inhibition of the IL-1β production in a dose-dependent manner when treated with trehalose [42].

Tumor necrosis factor-alpha (TNF-α) belongs to a family of inflammatory cytokines that utilize similar signaling pathways, including the activation of the transcription factor nuclear factor kappa B (NF-κB) and the initiation of apoptotic pathways [45,46]. These cytokines are associated with a diverse range of diseases, including cancer, arthritis, diabetes, atherosclerosis, and various inflammatory conditions.

Trehalose has been found to possess the ability to decrease TNF-a mRNA expression at 12 h in the study of Taya et al. (2009) mentioned above [42].

Yu et al. (2023) investigated the synthesis of non-protein inflammatory mediators, specifically eicosanoid PGE2 and the arginine metabolite nitric oxide (NO), along with the transcription of their respective converting enzyme genes (Cox-2 and iNOS) in a model using the RAW 264.7 cell line, originating from murine macrophages [43]. Trehalose exhibited notable effectiveness in inhibiting the transcription of Cox-2, a key enzyme involved in inflammation. Additionally, the substance significantly down-regulated the production of PGE2, a potent inflammatory mediator. Regarding iNOS transcription, trehalose exerted pronounced suppressive effects, which corresponded to a specific reduction in the synthesis of nitric oxide (NO), highlighting trehalose’s selective impact on inflammatory pathways.

As the information regarding trehalose and its effects on eicosanoids is relatively limited, further studies in that direction are needed.

#### 3.4.2. Suppression of NF-κB Signaling

The NF-κB transcription factor family plays a significant role in managing the immune response by regulating proinflammatory processes throughout the body. In its inactive state, NF-κB remains sequestered by IκB proteins found in the cytoplasm. When activated, IκB is degraded via proteasomes, leading to the liberation of NF-κB to translocate into the nucleus where its main function is as a transcription factor, followed by the expression of proinflammatory genes. The activation of NF-κB is generally triggered by various stimuli, predominantly consisting of pro-inflammatory cytokines and chemokines.

The downstream effects of NF-κB activation are highly specific to the cell type involved, which often culminates in the induction of proinflammatory cascades. In the central nervous system, the microglia serving as principal immune responders prominently upregulate the NF-kB in response to many pathological stimuli. This activation primes microglia for interaction with other cell types in the central nervous system, which can potentially trigger cellular death processes that can exacerbate disease pathology [47].

Trehalose inhibits the activation of NF-κB in vitro, resulting in the decreased transcription of pro-inflammatory genes. A study by Yu et al. from 2023, using the RAW 264.7 cell line, originating from murine macrophages, found that trehalose treatment did not alter the overall expression levels of NF-κB in comparison to both lipopolysaccharide (LPS)-treated and untreated (control) cells [43]. Notably, trehalose significantly reduced the expression of phosphorylated NF-κB relative to LPS-treated controls. These findings indicate that trehalose may inhibit LPS-induced inflammatory responses in vitro by modulating Toll-like receptor 4 (TLR4)-mediated NF-κB signaling.

While the reduction in phosphorylated NF-κB suggests an anti-inflammatory effect, the lack of alteration in overall NF-κB expression could imply that trehalose does not significantly affect the initiation of inflammatory responses (Table 3). This leads to an ambiguity: does trehalose primarily inhibit the activation of NF-κB or does it have broader effects on the inflammatory cascade?

### 3.5. Gut–Brain Signaling Modulation

The well-documented brain-gut axis refers to a bidirectional communication network between the central nervous system and the enteric nervous system. This network is mediated by neurons of the sympathetic and parasympathetic nervous systems, as well as by circulating hormones and various neuromodulatory molecules. Historically, it has been recognized as a mediator of gastrointestinal symptoms related to stress. However, interactions between the brain and gut go beyond the realms of stress, anxiety, or depression. They also include situations where both the brain and gut, along with their connection through the autonomic nervous system, are affected by the same pathological conditions, such as PD. The concept of this axis has now expanded to include the microbiota, forming the microbiota–gut–brain axis. Emerging evidence suggests that gut-resident bacteria can impact brain function, making the microbiome a potential target for diagnosing and treating a variety of disorders, including PD, AD, ALS, autism, stroke, depression, and drug addiction [48,49].

Trehalose has been investigated for its role in promoting gut health, particularly through its prebiotic effects [50]. Prebiotics like trehalose can selectively stimulate the growth and activity of beneficial gut bacteria, which in turn can influence gut–brain signaling via the gut microbiota–brain axis.

However, there are studies suggesting that trehalose can enhance the virulence of epidemic Clostridium difficile in a mouse model of infection [51], while other clinical studies summarized by Buckley et al. (2021) indicated no such correlation [52].

Further research is needed because these conflicting outcomes raise questions about the safety and efficacy of trehalose as a prebiotic. If it enhances the virulence of a harmful pathogen, it casts doubt on its beneficial effects on gut microbiota and overall gut health.

There is relatively limited research exploring the influence of trehalose on gut–brain signaling as compared to its effects on other aspects of health. However, some studies have suggested potential connections [11].

When animals consume trehalose, it undergoes hydrolysis by the trehalase enzyme in the gut. Although some trehalose may enter the bloodstream, its ability to reach the brain is limited by the blood–brain barrier. The primary effects of trehalose are likely to be observed predominantly within the gastrointestinal system. For instance, trehalose could potentially impact the gut microbiota by shielding it from harmful factors and stressors, thereby enhancing its resilience and overall survival [11]. This suggests that trehalose may play a beneficial role in gut health and microbial balance, influencing broader physiological outcomes through its actions at the gut level.

Evidence has increasingly shown that the gut microbiota can have a significant impact on various physiological systems including the central nervous system, which hints that trehalose might exert neuroprotective effects through modulating microbiota–gut–brain signaling [53]. In support of this, only the oral intake of trehalose and not an intraperitoneal injection led to autophagy induction in mouse brains, suggesting that the neuroprotective effects of trehalose require the involvement of the gastrointestinal system [54]. But besides these findings, the possibility that trehalose travels through the bloodstream and directly affects neurons in the brain cannot be ruled out entirely [11].

### 3.6. Additional Mechanisms Underlying the Neuroprotective Effects of Trehalose

In addition to its established function in enhancing autophagy, trehalose is implicated in influencing multiple biochemical pathways involved in secondary injury responses following traumatic brain injury (TBI) in C57BL/6 mice, notably including the regulation of brain metal homeostasis [55,56].

Essential metals such as copper, zinc, and iron are integral to fundamental biological processes like cellular metabolism, antioxidant defense mechanisms, and neurotransmission. However, dysregulation in their levels has been linked to exacerbated inflammatory responses, oxidative stress, and neuronal damage [57,58,59,60].

Recent studies suggest that trehalose can modulate the concentrations of these essential metals within the brain following TBI, potentially preventing their depletion or augmenting their levels. The precise molecular mechanisms by which trehalose exerts these effects on metal ion homeostasis remain a subject of ongoing investigation. Current hypotheses propose that trehalose may interact with regulatory proteins involved in metal storage, chelation processes, or transport mechanisms within brain cells. By adjusting the levels of these essential elements, trehalose may contribute to maintaining an optimal biochemical environment necessary for neuronal survival and proper cognitive function [61].

## 4. Discussion

Trehalose, a naturally occurring disaccharide, is known to have complex neuroprotective properties and covers several biological mechanisms essential to maintaining cell health and resilience, particularly in neurons and brains. One of the important ways that trehalose supports neuroprotection is by improving autophagy, a fundamental cell process that deals with the elimination of damaged proteins and organs. Trehalose achieves this by inhibiting the mTOR pathway, the central regulator of cell response to stress and food availability. By regulating mTOR signaling, trehalose promotes autophagy activation and facilitates cell component breakdown and recycling. This requirement is crucial to maintaining cell homeostasis and to mitigating the accumulation of toxic aggregates associated with neurodegenerative diseases.

One of the leading mechanisms by which trehalose promotes neuroprotection is by enhancing autophagy, an essential cellular process responsible for clearing damaged proteins and organelles from cells. Trehalose accomplishes this by inhibiting the mTOR pathway, a central regulator of cellular responses to stress and nutrient availability. By influencing and downregulating mTOR signaling, trehalose promotes the activation of autophagy, resulting in the breakdown and recycling of cellular components. This process is crucial for maintaining cellular homeostasis and reducing the buildup of toxic aggregates associated with neurodegenerative diseases.

Furthermore, trehalose activates AMPK, an energy-sensing enzyme that plays a main role in coordinating cellular energy metabolism and autophagy initiation. By this mechanism trehalose not only directly stimulates the ULK1 (Unc-51 Like Autophagy Activating Kinase 1) complex, a key initiator of autophagy, but also modulates other signaling pathways that contribute to autophagy flux. This dual mechanism enhances the efficiency of cellular “waste” clearance and supports cellular adaptation to stress conditions, thereby resulting in the promotion of neuronal survival and function.

In addition to its role in autophagy regulation, trehalose exerts direct effects on protein homeostasis by inhibiting the aggregation of misfolded proteins. Many neurodegenerative diseases, such as AD and PD, are characterized by the accumulation of misfolded proteins that form toxic aggregates. Trehalose interacts with these proteins through hydrogen bonding, stabilizing their structures and preventing the aberrant interactions that lead to pathological aggregation. By maintaining protein solubility and stability, trehalose helps alleviate protein aggregation-associated toxicity, consequently preserving neuronal integrity and function.

Moreover, trehalose acts as an osmoprotectant, safeguarding neurons against osmotic stress by stabilizing cellular membranes and protein structures. This protective function is particularly important under conditions of dehydration or exposure to high osmolarity environments. In such environments, trehalose replaces water molecules in protein hydration shells and interacts with phospholipids to maintain membrane integrity. By reducing membrane permeability and preventing cellular deformation, trehalose contributes to neuronal resilience against osmotic challenges, thereby supporting overall cellular health.

Beyond its roles in cellular maintenance and stress response, trehalose exhibits anti-inflammatory properties that are beneficial in neurodegenerative damage. It modulates immune responses by suppressing the production of pro-inflammatory cytokines such as IL-1β and TNF-α and inhibiting NF-κB signaling pathways. By dampening the excessive inflammatory responses implicated in neurodegeneration, trehalose helps mitigate neuroinflammation and its detrimental effects on neuronal function and survival.

Furthermore, emerging research suggests that trehalose may impact neurological health indirectly through interactions with the gut microbiota and the gut–brain axis. Changes in gut microbial composition and function can influence systemic inflammation, potentially impacting brain function and neurodegenerative processes. Trehalose’s ability to modulate gut microbiota and inflammatory responses highlights its broader therapeutic potential in supporting brain health and ameliorating neurodegenerative diseases.

Recent research has substantially advanced our understanding of trehalose’s neuroprotective effects by proposing a novel hypothesis that highlights its involvement in essential metal homeostasis. According to this emerging hypothesis, trehalose may be instrumental in regulating and maintaining the balance of critical metals such as copper, zinc, and iron. These metals are vital for various cellular processes, including enzymatic functions, antioxidant defense, and neurotransmission. The hypothesis suggests that by modulating the levels and distribution of these essential metals, trehalose may mitigate dysregulation-related pathologies, thereby enhancing its neuroprotective effects. This perspective provides a promising new avenue for exploring how trehalose contributes to neuronal health and resilience.

Despite the promising neuroprotective properties of trehalose, several important limitations and challenges need to be addressed. One of the primary concerns is its relatively poor bioavailability and the difficulty of achieving therapeutically effective concentrations in the central nervous system through oral administration. Trehalose’s large molecular size and limited permeability across the blood–brain barrier present significant hurdles for systemic administration, potentially reducing its effectiveness in treating neurological conditions. Additionally, while studies have shown beneficial effects, there are unresolved issues regarding the long-term safety of trehalose. Potential side effects, such as gastrointestinal discomfort and possible interactions with other medications, could impact patient tolerance and adherence. Furthermore, the optimal dosing strategies for trehalose are not yet well-defined, and variations in individual responses could complicate treatment regimens. To maximize the therapeutic potential of trehalose, future research must focus on developing advanced drug delivery systems, optimizing dosing protocols, and thoroughly assessing its safety profile. Addressing these challenges will be crucial for translating the promising preclinical findings into effective clinical applications.

The findings discussed in the paper underscore the significant potential of trehalose as a therapeutic agent for various neurodegenerative diseases. Trehalose’s effects on autophagy and protein aggregation are highly relevant to the pathophysiology of AD, PD, and HD. In AD, trehalose’s ability to enhance autophagic clearance could be instrumental in reducing the accumulation of amyloid-beta plaques and hyperphosphorylated tau tangles, which are hallmark features of the disease. For PD, trehalose’s role in improving lysosomal function may help in mitigating the aggregation of alpha-synuclein, a key protein implicated in the disease’s progression. In the context of HD, trehalose might offer therapeutic benefits by influencing the aggregation of mutant huntingtin protein, which is critical for the disease’s manifestation. These proposed mechanisms align with the cellular dysfunctions observed in these neurodegenerative conditions. However, it is crucial to emphasize that while the current evidence suggests a promising role for trehalose, the data are preliminary. Comprehensive clinical trials and more studies are necessary to confirm its effectiveness and elucidate the precise pathways through which trehalose exerts its effects. Continued research will be essential in establishing trehalose as a viable therapeutic option and in understanding its full potential in the treatment of neurodegenerative diseases.

In summary, trehalose stands out as a promising candidate for neuroprotective interventions due to its diverse mechanisms of action. From promoting autophagy and inhibiting protein aggregation to acting as an osmoprotectant and modulating inflammatory responses, trehalose offers comprehensive support for neuronal resilience and function. Continued research into its therapeutic applications holds promise for addressing neurodegenerative diseases and other neurological disorders where maintaining cellular health and mitigating stress responses are crucial for optimal brain function.

## Figures and Tables

**Table 1:** Trehalose’s mechanisms of autophagy involvement.

Autophagy Mechanism	Detailed Mechanism of Autophagy Activation	Reference
mTOR^1^ pathway involvement	Interaction with mTORC1^2^	[24]
	Stress-response activation AMPK^3^ involvement	[16] [22,23]
mTOR-independent	Inducing autophagy without influencing the mTOR pathway	[13,19,25]
TFEB^4^ activation	Influencing nutrient-sensing pathways that regulate TFEB Facilitating lysosomal calcium release	[16] [16]
	Increased synthesis of lysosomal proteins	[9]

mTOR^1^—mechanistic target of rapamycin; mTORC1^2^—mTOR complex 1; AMPK^3^—AMP-activated protein kinase; TFEB^4^—transcription factor EB.

**Table 2:** Trehalose’s mechanisms of inhibition of misfolded protein aggregation.

Detailed Mechanism	Reference
stabilizing protein molecules by forming hydrogen bonds	[30,31]
stabilization of hydration shells and water replacement	[34]
protection against heat	[6,31]
reduction in misfolded protein burden	[7]
interaction with amyloidogenic proteins	[13,33]

**Table 3:** Trehalose’s mechanisms of anti-inflammatory activity.

Detailed Mechanism	Reference
inhibition of pro-inflammatory cytokine activity	[42,43]
suppression of ^1^NF-κB signaling	[43]
suppression of inflammatory mediator production	[43]

^1^NF-κB—nuclear factor kappa B.

## Data Availability

Not applicable.
